# Pair-Wise Regulation of Convergence and Extension Cell Movements by Four Phosphatases via RhoA

**DOI:** 10.1371/journal.pone.0035913

**Published:** 2012-04-24

**Authors:** Mark van Eekelen, Vincent Runtuwene, Wouter Masselink, Jeroen den Hertog

**Affiliations:** 1 Hubrecht Institute and University Medical Center Utrecht, Utrecht, the Netherlands; 2 Institute of Biology, Leiden, the Netherlands; Institute of Science and Technology Austria, Austria

## Abstract

Various signaling pathways regulate shaping of the main body axis during early vertebrate development. Here, we focused on the role of protein-tyrosine phosphatase signaling in convergence and extension cell movements. We identified Ptpn20 as a structural paralogue of PTP-BL and both phosphatases were required for normal gastrulation cell movements. Interestingly, knockdowns of PTP-BL and Ptpn20 evoked similar developmental defects as knockdown of RPTPα and PTPε. Co-knockdown of RPTPα and PTP-BL, but not Ptpn20, had synergistic effects and conversely, PTPε and Ptpn20, but not PTP-BL, cooperated, demonstrating the specificity of our approach. RPTPα and PTPε knockdowns were rescued by constitutively active RhoA, whereas PTP-BL and Ptpn20 knockdowns were rescued by dominant negative RhoA. Consistently, RPTPα and PTP-BL had opposite effects on RhoA activation, both in a PTP-dependent manner. Downstream of the PTPs, we identified NGEF and Arhgap29, regulating RhoA activation and inactivation, respectively, in convergence and extension cell movements. We propose a model in which two phosphatases activate RhoA and two phosphatases inhibit RhoA, resulting in proper cell polarization and normal convergence and extension cell movements.

## Introduction

Early vertebrate embryonic development is characterized by three processes, cell proliferation, differentiation and migration. In order to form the basic body plan and - at a later time-point - organs, cells will not only need to differentiate to become the proper cell type, but they will need to be at the right place at the right time. In vertebrates the earliest two processes conducted by cell migration are the formation of the three germ layers during gastrulation by epiboly and internalization (or ingression/emboly, depending on the organism), and the formation of the medio-lateral body axis by convergence and extension (C/E) cell movements [1], [2]. C/E cell movements require cells of the axial and paraxial mesoderm and neurectoderm to polarize and elongate in their direction of movement. These cells migrate towards the dorsal midline and participate in a process called intercalation in order to extend the body axis. C/E cell movements are highly coordinated, using lateral lamellipodia to actively and directionally crawl between neighboring cells towards the midline to align there. Impaired C/E cell movements result in shorter and wider embryos, which can be accounted for by fewer cells reaching the dorsal midline and decreased intercalation. Additional phenotypes of C/E defects constitute neural tube defects and cyclopia [1], [3], [4], [5], [6], [7], [8], [9].

Although the mechanisms of gastrulation cell movements have been well described, the underlying molecular regulation remains elusive. Over the past years many proteins have been reported to contribute to C/E cell movements. C/E cell movements are expected to be affected by proteins involved in cell polarity, migration, adhesion and more, explaining why many mutant/knockdown phenotypes give rise to C/E defects. Several signaling pathways are known to participate in proper C/E cell movements, like Bmp signaling [10], [11], PDGF-PI3K signaling [12], [13], [14], Jak-Stat signaling [15], [16] and Eph-ephrin signaling [17], [18], [19], but the most extensively described is the non-canonical wnt/Planar Cell Polarity (PCP) signaling pathway [3], [20], [21], [22], [23], [24], [25], [26], [27]. The PCP pathway was first identified in *Drosophila* where organization of wing epithelial hairs is regulated by this pathway. The term PCP is used to describe the organization of cells and their components within a plane, usually an epithelial layer. In flies, the PCP pathway regulates the asymmetric localization of several core PCP proteins like Vangl, Pk, Fz, Dsh and Dgo [28], [29], [30], [31], [32], [33], which in turn regulates the morphology of the wing epithelium with a wing-hair at the distal tip of each cell. Other epithelial structures organized by PCP signaling are the drosophila eye and vertebrate hair cells in the cochlea [34], [35]. Although the function of all the core PCP components is not completely understood, the main function seems to be regulating cell-cell communication in order to organize structure. In vertebrates, a major part of this pathway consists of the non-canonical wnt signaling pathway. Non-canonical Wnt signaling is β-catenin independent and involves Wnt4/5a/7a/11, Fz3/6/7, Dvl1/2/3 and Pk1/2 to activate the two main downstream components, RhoA and Rac1 [36], [37], [38].

PTPs play an import role in signal transduction in concert with their enzymatic counterparts, the protein-tyrosine kinases (PTKs). RPTPσ and LAR for example have an important role in neuronal development [39], [40], [41], while CD45 has a critical function in immune cell regulation [42]. The role of Shp2 has been extensively studied in mouse and zebrafish, as activating and inactivating mutations lead to Noonan and LEOPARD syndrome in humans [43]. We have shown previously that Noonan and LEOPARD associated mutations in Shp2 confer C/E cell movement defects in zebrafish [44]. We also reported two other PTPs in C/E cell movements, RPTPα and PTPε [45], that mediate their effects by activation of RhoA through the Src family kinases (SFKs) Fyn and Yes.

Here, we show that four PTPs are involved in regulating cell polarity and C/E cell movements. RPTPα and PTPε activate RhoA through the Fyn and Yes SFKs, and PTP-BL and Ptpn20 inhibit RhoA activity. Co-knockdown experiments indicate that these four PTPs work in pairs. The Rho-GEF, NGEF, acts downstream of RPTPα and PTPε, and the Rho-GAP, Arhgap29 (Parg1), downstream of PTP-BL and Ptpn20. We suggest a model where RhoA is activated following recruitment of NGEF upon RPTPα/PTPε and Fyn/Yes mediated stimulation and RhoA activity is inhibited following recruitment of Arhgap29 upon PTP-BL/Ptpn20 mediated repression. Based on these results, we conclude that normal activation and inhibition of RhoA is required for proper cell polarization and normal C/E cell movements.

## Results

### Identification of *Ptpn20* as a homologue of PTP-BL

**Figure 1:**
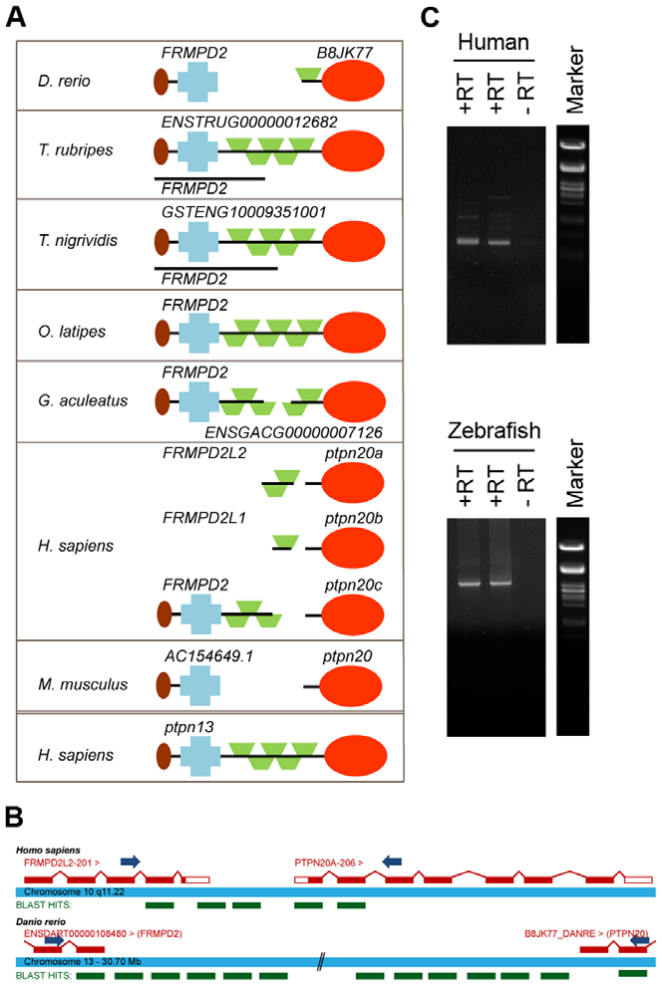
Identification of *ptpn20* as a homologue of *ptpn13*. (a) Protein structures are shown encoded by *ptpn20* homologue and the immediately 5′ upstream *FRMPD2*, as currently annotated in five fish genomes, the human genome and the mouse genome. In some cases like Fugu and Tetraodon a single known coding transcript exists besides separate transcripts encoding the PTP domain and the “FRMPD" part. For comparison the protein structure encoded by human *ptpn13* (PTPBL) is added below. (b) Primers were designed as indicated, leaving approximately 100 bp known coding sequence for the purpose of alignment of generated sequences. PCR products with forward primers on the second to last known exon of human and zebrafish *FRMPD2* and reverse oligos on the second exon of *PTPN20*. A schematic representation of retrieved sequences blasted to the genome are indicated in green (not to scale). (c) Generated PCR products on human (top) and zebrafish (bottom) cDNA libraries using the described primer sets. Generated band sizes are consistent with expected values based on homology with the *ptpn13* gene.

We recently identified all protein tyrosine phosphatase (PTP) genes in the zebrafish genome by blasting the individual PTP domains of human genes against the zebrafish genome (Zv8, Ensembl) [46]. We compared the genes we identified with four other fish genomes available (medaka, fugu, stickleback and tetraodon) to evaluate our findings (Fig. S1 and Table S1). Although these fish genomes were not completely annotated, in general they were more complete than the zebrafish genome, and missing PTP encoding genes could easily be identified by blasting. When aligning several candidate genes for *ptpn20* we noticed that some fish genes were annotated with different names and protein structures (Fig. 1a). Having a closer look at the *ptpn20* candidate genes, we found that in *Oryzia latipes* this gene was annotated as *frmpd2* and in *Tetraodon nigrividis* as *GSTENG10009351001*, both bearing remarkable resemblance to the structure of the human *PTPN13* gene encoding PTP-BL (also known as PTP-BAS, PTP-L1 or FAP1). Interestingly, the PTP domain of *ptpn13* has the highest sequence homology to the PTP domain of *ptpn20*. Upon further investigation we found a gene named *frmpd2* or a gene with similar structure to the 5′ side of *ptpn20* in all species, including the human genome which according to the Ensembl database contains 3 copies of *ptpn20*; *ptpn20a*, *ptpn20b* and *ptpn20c*, all accompanied with their own *frmpd2-(like)* gene. We hypothesized that *frmpd2* and *ptpn20* might in fact be a single gene with structural resemblance to *ptpn13*, like *frmpd2* in *O. latipes*. In order to test this hypothesis, we generated cDNA from zebrafish embryos and HEK293 cells using reverse transcription (RT) and designed forward primers on the second to last known coding exon of *frmpd2* and reverse primers on the second known coding exon of *ptpn20* (Fig. 1b). We performed PCR using these primer sets and generated PCR products indicating that single transcripts containing *frmpd2* and *ptpn20* coding sequence exist (Fig. 1c). These PCR products were sequenced and these were blasted back to the zebrafish and human genome, resulting in identification of the missing exons connecting the two transcripts, confirming the existence of a single *ptpn13*-like *ptpn20* transcript (Fig. 1b). Full length *ptpn20* transcript encodes a protein with a FERM domain, KIND domain, 5 PDZ domains and a PTP domain, similar to PTP-BL. We conclude - based on their structural resemblance - that Ptpn20 and PTP-BL are paralogues, which is reflected by the high sequence similarity between the PTP domains of PTP-BL and Ptpn20.

### *Ptpn13*, *ptpn20*, *ptpra* or *ptpre* knockdown results in defective C/E cell movements and cell polarization

**Figure 2:**
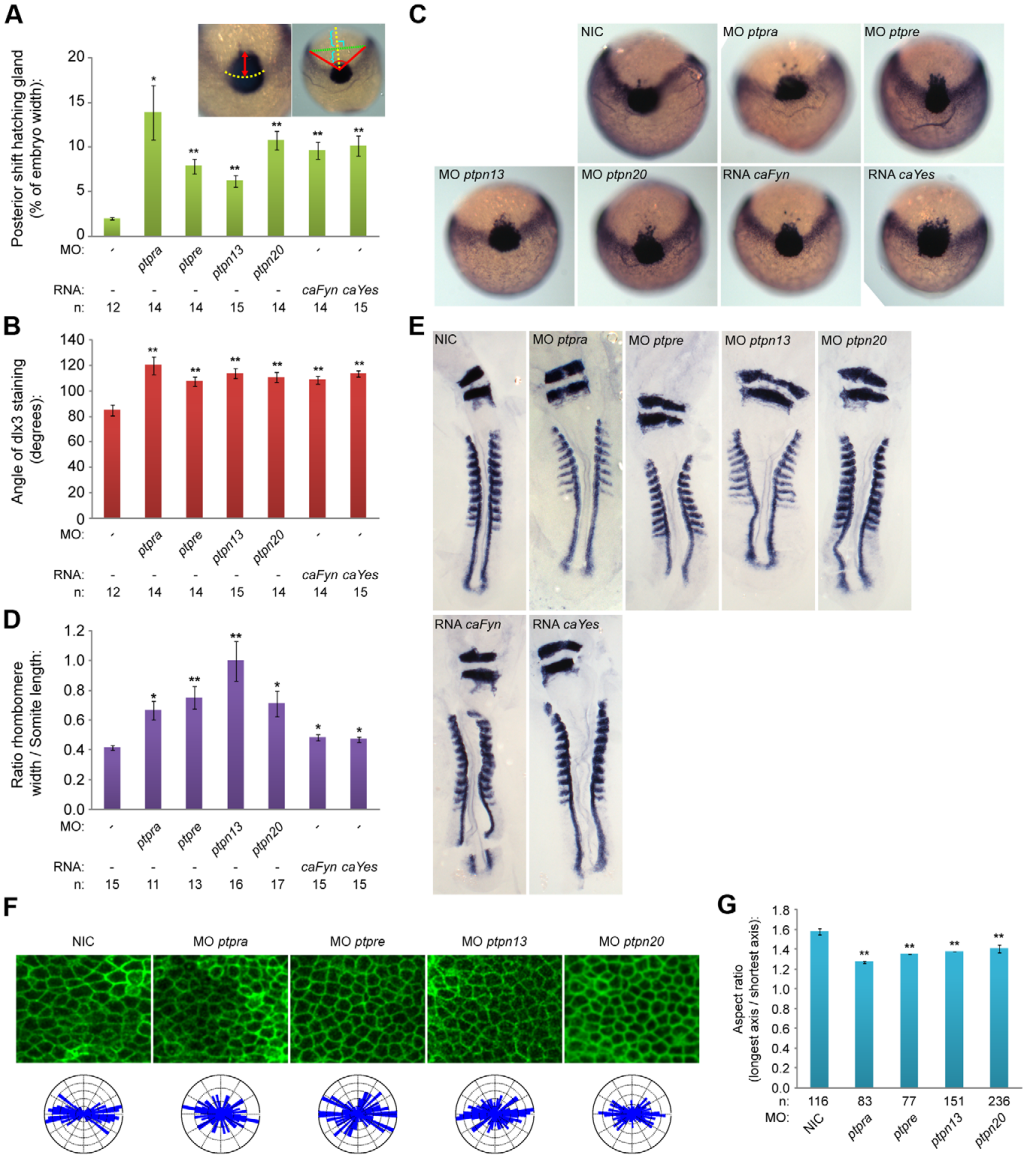
PTP knockdowns affect C/E and cell polarization. (a) Zebrafish embryos were microinjected with morpholinos (high concentration) targeting the different phosphatase genes or RNA constructs encoding constitutively active forms of Fyn or Yes at the one cell stage and grown to 1 somite stage. Embryos were fixed and stained for *dlx3* and *hgg1* expression using whole mount *in situ* hybridization, staining the precursors of the hatching gland (*hgg1*) and the edge of the neural plate (*dlx3*). Posterior shift of the hatching gland and angle of *dlx3* staining are measured as shown in inset, the results are plotted in (a) and (b). Pictures of representative embryos used in the quantifications in (a) and (b) are shown in (c). Embryos were microinjected using the same conditions as described above and grown to 8–9 somite stage. Embryos were fixed and stained for *krox20* and *myod* using whole mount *in situ* hybridization. *Krox20* stains rhombomere 3 and 5, while *myod* stains the somites. Resulting staining patterns were used to quantify width to ratio by measuring rhombomere width (*krox20*) and somite length (8 somites, *myod*). Ratios are plotted in (d), representative embryos are depicted in (e). (f) Zebrafish embryos were micro-injected using the constructs described above, co-injected with RNA encoding YFP-caax and RNA encoding mCherry-H2B at the one cell stage and mounted at shield stage. Embryos were imaged over time at the presomitic mesoderm, representative areas of presomitic mesoderm for each condition are shown. Resulting images were analyzed for cell shape (aspect ratio) by dividing the length of the longest axis by the length of the shortest axis for each cell, average aspect ratios are plotted in (g). The distribution of angles of the longest axis towards the dorsal midline were plotted in rose-plots and shown in (f; bottom). All error bars are standard error of the mean. Student t-tests were performed with non-injected control; no asterisk indicates P>0.05, * indicates 0.05>P>0.001 and ** indicates P<0.001.

PTP-BL is a large multi-domain protein containing a FERM, KIND, 5 PDZ and a PTP domain, suggesting a role as a scaffold protein since all except the PTP domain play a role in protein-protein interactions. Indeed many binding partners have been described, suggesting an inhibiting role in Fas-mediated apoptosis [47], [48] and a role in SFK dependent phosphorylation of ephrin-B [49], [50]. We designed splice donor morpholinos targeting the active site of the PTP domain and demonstrated that *ptpn13* induced C/E defects. We performed *in situ* hybridization with probes staining *dlx3*, *hgg1*, *krox20* and *myod*, all well-established markers for C/E cell movements [51], [52]. *Dlx3* stains the edge of the neural plate which in the case of impaired convergence will be wider, while *hgg1* stains the precursors of the hatching gland, which in the case of defective extension movements will be shifted posteriorly. We fixed embryos at the one somite stage and performed whole mount *in situ* hybridization. By quantifying the angle of *dlx3* staining and the length of the anterior shift of *hgg1* staining as indicated (Fig. 2a, inset), we found that knockdown of *ptpn13* significantly affects C/E cell movements (Fig. 2a–c). The phenotype observed in *ptpn13* knockdown embryos was fully rescued by co-injection of mouse *ptpn13* RNA (Fig. S2). Interestingly, ptpn20 knockdown induced similar C/E cell movement defects (Fig. 2a–c). Moreover, *ptpra* and *ptpre* knockdowns also induced C/E defects (Fig. 2a–c). To assess C/E defects in an independent manner, we performed *in situ* hybridization experiments with probes for *krox20*, which stains rhombomeres 3 and 5, and *myod*, which stains the somites. In case of defective C/E movements the rhombomeres will be wider (reduced convergence) and the length of 8 somites will be shorter (reduced extension). Defects can be quantified by calculating the ratio of the width of rhombomere 3/the length of 8 somites. Using this read-out, we again established that knockdown of *ptpn13*, *ptpn20*, *ptpra* and *ptpre* induced significant C/E cell migration defects (Fig. 2d,e)

We previously showed that RPTPα and PTPε function in C/E cell movements by activation of the SFKs, Fyn and Yes [45], [46]. We assessed the effects of expression of constitutively active mutants of Fyn and Yes that harbor point mutations (Tyr to Phe) in their inhibitory C-terminal phosphorylation sites on C/E cell movements. As expected, injection of constitutively active variants of *fyn* and *yes* mRNA (*caFyn* and *caYes*) also induced C/E cell movement defects as assessed using the dlx3/hgg1 and krox20/myod markers (Fig. 2a–e).

C/E cell movement defects can result from defective cell polarization, resulting in less elongated cells with reduced polarization towards the dorsal midline. In order to investigate if cell polarization is causing the observed phenotypes, we determined the shapes of dorsally migrating presomitic cells as described before [45]. Wildtype or knockdown embryos were (co-)injected with YFP-caax mRNA and mCherry-H2B to label the cell membrane and nuclei, respectively. We imaged cell shapes in the presomitic mesoderm (Fig. 2f) and determined the cell elongation by analyzing the membrane marker YFP-caax and calculated the aspect ratio (the longest axis divided by the shortest axis). This aspect ratio is directly proportional to cell polarization and is significantly reduced upon knockdown of *ptpn13*, *ptpn20*, *ptpra* and *ptpre* (Fig. 2g). Imaging of presomitic mesoderm cells also provided us with a means to assess the angle that single cells make towards the dorsal midline. These angles were plotted in rose diagrams, and indicate that *ptpn13*, *ptpn20*, *ptpra* and *ptpre* knockdown results in more random distribution of the cell axis and less elongated presomitic cells, compared to wildtype embryos (Fig. 2f). Taken together, we show that RPTPα, PTPε, PTP-BL and Ptpn20 are involved in C/E cell movements by regulating cell polarity.

### *Ptpn20* and *ptpn13* show redundancy, and function together with *ptpra* and *ptpre*

**Figure 3:**
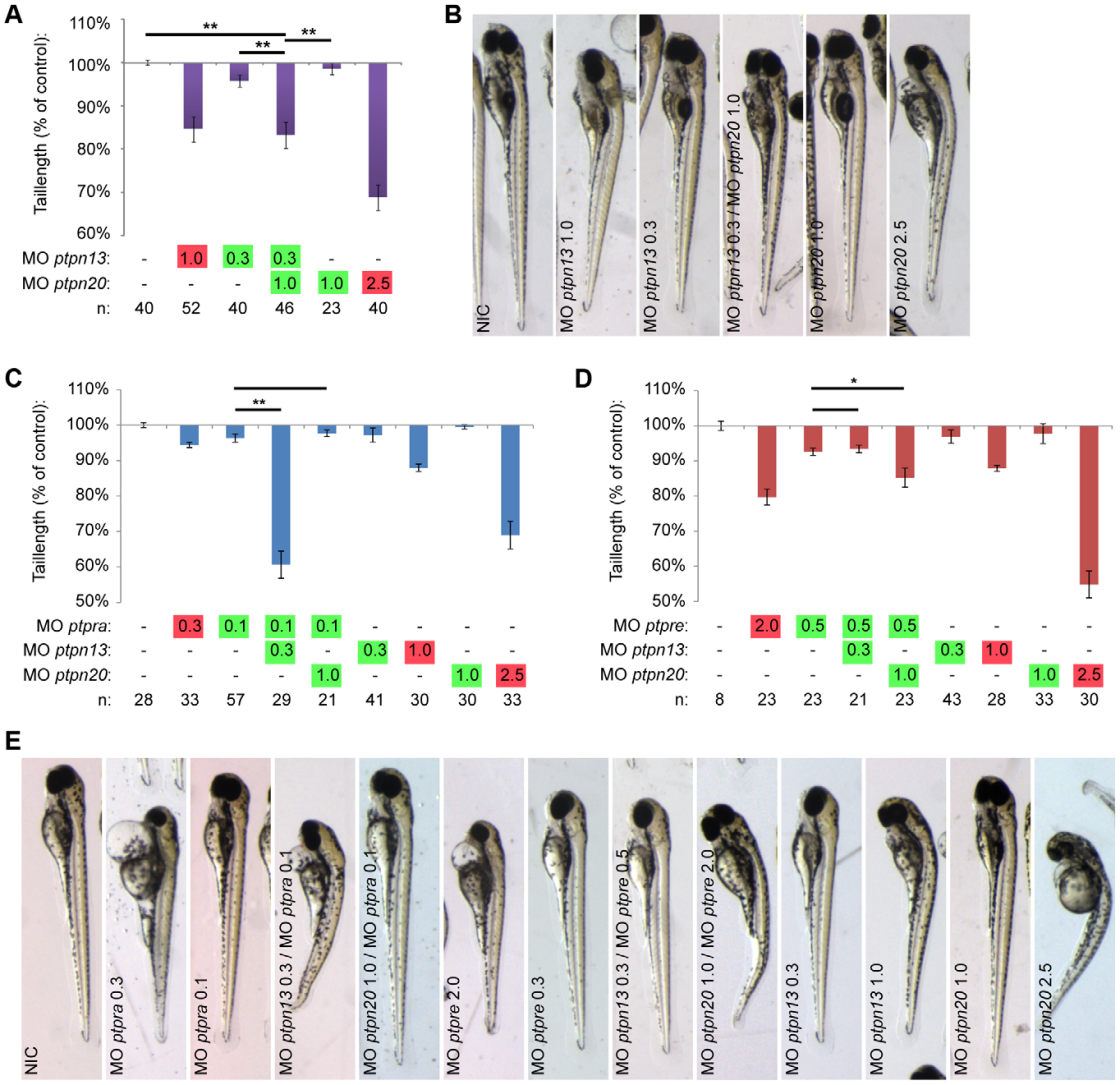
*Ptpn13* and *ptpn20* cooperate with each other and with *ptpra* and *ptpre*. Morpholinos targeting *ptpn13* and *ptpn20* were injected in the zebrafish at the one cell stage, and concentrations were titrated down until no phenotype was observed. Normal (red), low (green) concentrations and combined low concentrations of *ptpn13* and *ptpn20* morpholino were micro-injected and embryos were grown to 3dpf under normal conditions. Pictures were taken from all embryos and tails were measured using ImageJ imaging software, from the yolk to the tip of the tail, and compared to non-injected control. Average tail length compared to non-injected control is plotted as a percentage deviating from 100% in (a) and representative fish are shown for each condition in (b). Zebrafish embryos were microinjected as described above, using low concentration combined knockdown of *ptpra* with either *ptpn13* or *ptpn20*, or *ptpre* with either *ptpn13* or *ptpn20* and tail lengths are plotted in (c) and (d). (e) Shown are representative fish from the experiments depicted in (c) and (d). All error bars are standard error of the mean. Student t-test was performed where indicated; no asterisk indicates P>0.05, * indicates 0.05>P>0.001 and ** indicates P<0.001. Morpholino concentrations are color coded: red for “full" knockdown, giving full phenotype without being toxic and green for “low" concentration, giving no observable phenotype.

Knockdown of all four PTPs induced C/E cell movement defects (Fig. 2). One of the hallmarks of C/E cell migration defects during gastrulation is the severely shortened embryo body axis at 3days post fertilization (dpf) (Fig. 3a). The tail length at 3 dpf directly correlates to the severity of C/E related phenotypes [45]. We measured the tail length as an easy and unbiased method to quantify C/E defects. Given the similarity in phenotypes and the structural similarity between PTP-BL and Ptpn20, we proceeded to investigate whether combined knockdowns act synergistically. To this end, we titrated morpholinos down until no obvious phenotype was observed and these low doses of morpholinos were combined. Genes functioning in the same pathway will reconstitute the original (full dosage) phenotype, in this case shorter fish embryos, like we have shown previously in combined *ptpra* and *ptpre* knockdown [45]. Although tail length by itself does not discriminate between different possible processes that could underlie defects in body axis extension, we believe that - combined with detailed analysis of C/E cell movement defects in the full knockdowns - this method accurately identifies components of the same pathway and is suitable for screening purposes. For convenience, low dosage morpholino concentrations will be indicated in figures throughout this manuscript in green whereas full dosage morpholino concentrations are indicated in red. Using this method, we found that knockdown of either *ptpn13* or *ptpn20* induced shortened embryo body axes (Fig. 3a,b). Low doses of these morpholinos did not induce phenotypes by themselves. Combined low dose *ptpn13* and *ptpn20* knockdown induced a similar phenotype as high dose knockdown of either *ptpn13* or *ptpn20*, suggesting that *ptpn13* and *ptpn20* knockdowns acted synergistically.

We have previously described a similar phenotype in *ptpra* and *ptpre* knockdown zebrafish, as well as in *ptpra^−/−^* fish lines. Therefore we decided to investigate if these four PTPs might function in the same pathway by using low dose combined knockdown of *ptpn13* or *ptpn20* with either *ptpra* or *ptpre*. As a readout, we investigated if these combinations resulted in reconstitution of the shorter phenotype. We found that combining *ptpn13* and *ptpra* or *ptpn20* and *ptpre* knockdown specifically reconstituted shorter tail length phenotypes. Interestingly combining *ptpn13* and *ptpre* or *ptpn20* and *ptpra* did not induce shorter fish (Fig. 3c–e). These results indicate that these PTPs acted in pairs and illustrate that our analyses were specific in that not just any pair of PTP morpholinos induced tail defects.

### C/E cell movement defects are caused by defective RhoA regulation

**Figure 4:**
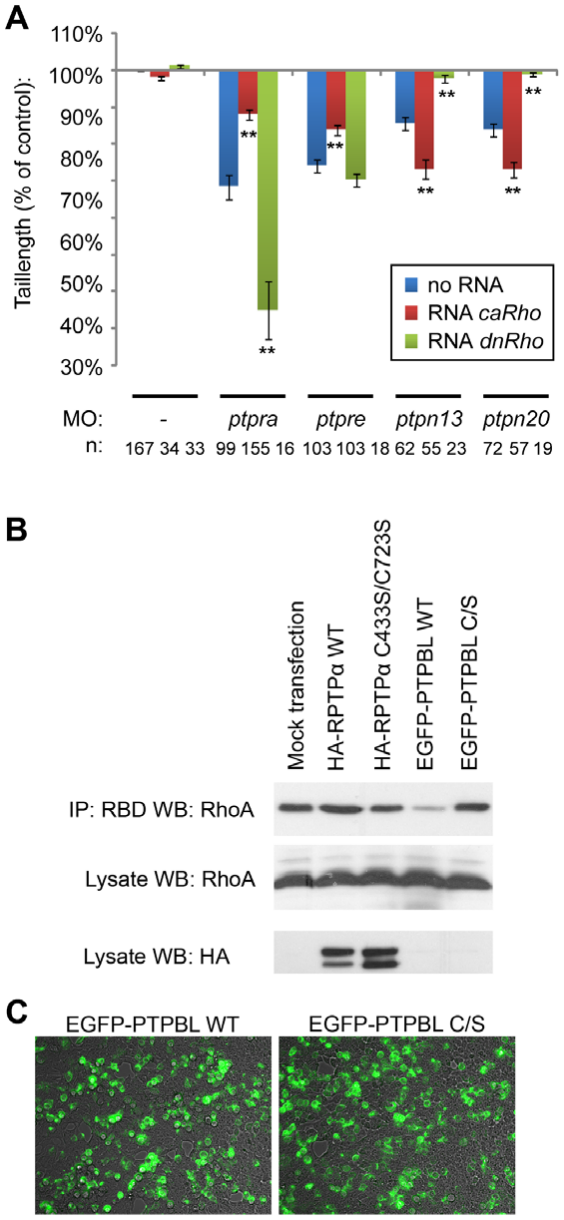
PTPs affect RhoA activation. (a) PTP knockdowns are rescued by active or dominant negative RhoA. Embryos were micro-injected at the one cell stage using morpholinos (high concentration) targeting the indicated genes together with no RNA, RNA encoding constitutively active RhoA (3 pg/embryo) or RNA encoding dominant negative RhoA (20 pg/embryo). Fish were grown to 3dpf and tail lengths were measured. Average tail length compared to non-injected control is plotted. All error bars are standard error of the mean. Student t-tests were performed between morpholino knockdown and RNA co-injections with the same morpholino; no asterisk indicates P>0.05, * indicates 0.05>P>0.001 and ** indicates P<0.001. (b) Direct effects of PTPs on RhoA activation. HEK293T cells were either mock transfected or transfected with HA-RPTPα-WT, HA-RPTPα-C433S/C723S, EGFP-PTPBL-WT or EGFP-PTPBL-C/S. Cells were lysed and GTP-bound Rho was selectively precipitated using Rhotekin RBD-beads. The beads were washed and precipitated RhoA was detected using a RhoA-specific antibody (top panel). Total RhoA (middle panel) and transfected HA-RPTPα (bottom panel) was monitored in lysate by immunoblotting. (c) Expression of EGFP-PTPBL-WT or EGFP-PTPBL-C/S was monitored by fluorescence microscopy. Representative images are depicted here.

RhoA has been shown to play a major role in cell polarization [5], [6] and RhoA is activated during cell movements in response to Wnt11 and Wnt5a. Shp2, RPTPα, PTPε, Fyn and Yes also signal to RhoA in C/E [45], [53], [54]. In order to test whether defective RhoA regulation is at the basis of the cell polarization defects observed here, we co-injected *ptpra*, *ptpre*, *ptpn13* and *ptpn20* morpholinos with either RNA encoding constitutively active *rhoa* (*caRhoA*) or dominant negative *rhoa* (*dnRhoA*). We used tail length at 3dpf as readout to see if co-injections were able to rescue or further increase the knockdown phenotypes. As described before [45] *caRhoA* mRNA can rescue *ptpra* and *ptpre* knockdown. As expected, co-injection of *dnRhoA* mRNA in *ptpra* and *ptpre* knockdown embryos increased the phenotype. Surprisingly, co-injection of *ptpn13* and *ptpn20* morpholinos with *caRhoA* mRNA worsened the phenotype and co-injection of *dnRhoA* mRNA with *ptpn13* and *ptpn20* morpholinos rescued the phenotype (Fig. 4, Fig. S3). Our results suggest that RPTPα and PTPε have an activating effect on RhoA, whereas PTP-BL and Ptpn20 inhibit RhoA activity. To assess the effects of RPTPα and PTP-BL on RhoA activation directly, we expressed RPTPα or PTP-BL in HEK293T cells and selectively precipitated GTP-bound Rho using the Rhotekin Rho-binding domain. A higher proportion of RhoA was precipitated upon expression of RPTPα, compared to mock-transfected cells (Fig. 4b). In contrast, a lower proportion of GTP-bound RhoA was precipitated upon expression of PTP-BL. As controls, we expressed catalytically inactive RPTPα or PTP-BL with Cys to Ser mutations in their catalytic sites to a similar extent as their wild type counterparts as assessed by immunoblotting or fluorescence microscopy (Fig. 4b,c). Expression of either catalytically inactive PTP did not affect the proportion of GTP-bound RhoA that was precipitated in these assays (Fig. 4b). These results indicate that RPTPα activates RhoA, whereas PTP-BL inhibits RhoA, which is consistent with the observed effects in zebrafish embryos.

### RhoA is activated by NGEF and inactivated by Arhgap29

**Figure 5:**
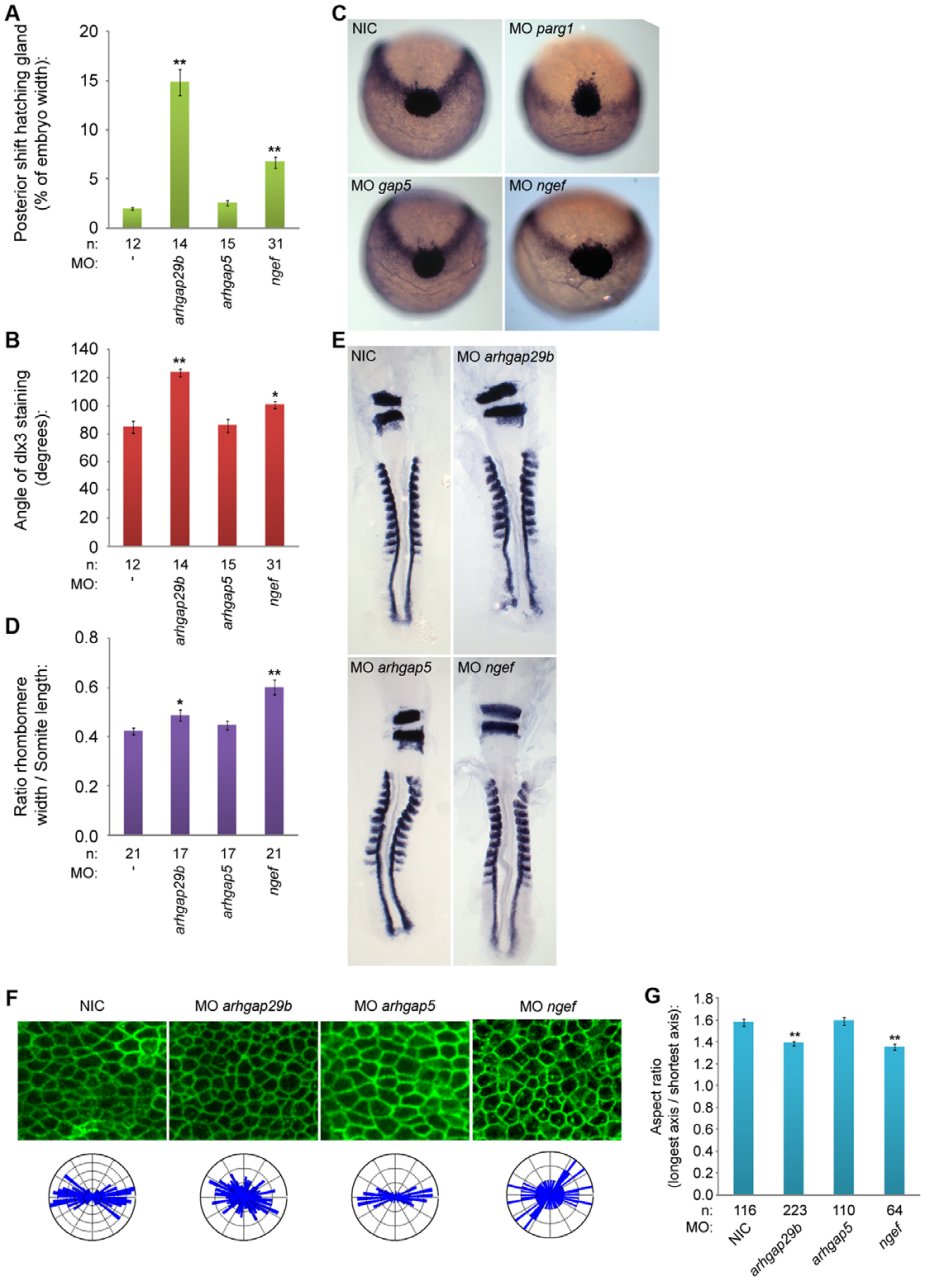
Knockdown of *ngef* or *arhgap29b* induces C/E cell movement and cell polarization defects. Zebrafish embryos were microinjected with morpholinos (high concentration) targeting *arhgap29b*, *arhgap5* or *ngef* at the one cell stage and grown to 1 somite stage. Embryos were fixed and stained for *dlx3* and *hgg1* expression using whole mount *in situ* hybridization. Posterior shift of the hatching gland and angle of *dlx3* staining are measured as in Fig. 3. (a,b). Representative embryos are shown in (c). Embryos were grown to 8–9 somite stage, fixed and stained for *krox20* and *myod*. Rhombomere width (*krox20*) and somite length (8 somites, *myod*) ratios are plotted in (d); representative embryos are depicted in (e). (f) Representative areas of presomitic mesoderm for the indicated conditions were analyzed for cell shape and the distribution of angles of the longest axis towards the dorsal midline was plotted in rose-plots (f; bottom); aspect ratio plotted in (g). All error bars are standard error of the mean. Student t-tests were performed with non-injected control; no asterisk indicates P>0.05, * indicates 0.05>P>0.001 and ** indicates P<0.001.

RhoA is a member of the Rho family GTPases that is activated by Rho guanine nucleotide exchange factors (Rho-GEFs) and inactivated by Rho GTPase-activating proteins (Rho-GAPs). Of the many Rho-GEFs and Rho-GAPS described in the literature it is not known which have a role in C/E cell movements. We decided to investigate Arhgap29, which is also known as Parg1 (PTP-BL associated Rho-GAP1), that has previously been shown to bind directly to PTP-BL [55]. We used a non-related Rho-GAP, Arhgap5 (also known as Gap5) as a control. Knockdown of *arhgap29b* induced C/E cell movement defects as assessed by whole mount *in situ* hybridization using *dlx3*/*hgg1* and *krox20*/*myod* as markers (Fig. 5a–e). *Arhgap5* knockdown did not induce C/E cell movement defects, indicating that the *arhgap29* knockdown phenotype was specific (Fig. 5a–e). NGEF, also known as Ephexin/Arhgef27, is tightly regulated by tyrosine (de)phosphorylation [56] and hence it is a good candidate Rho-GEF to mediate the effects of PTPs. Knockdown of *arhgef27* induced C/E cell movement defects in zebrafish embryos (Fig. 5a–e). We analyzed cell polarization in Rho-GAP and Rho-GEF knockdown embryos and observed decreased cell elongation specifically in *ngef* and *arhgap29b* knockdown embryos, but not in *arhgap5* knockdown embryos (Fig. 5f–g). These data are consistent with NGEF and Arhgap29 acting in cell polarization and C/E cell movements.

**Figure 6:**
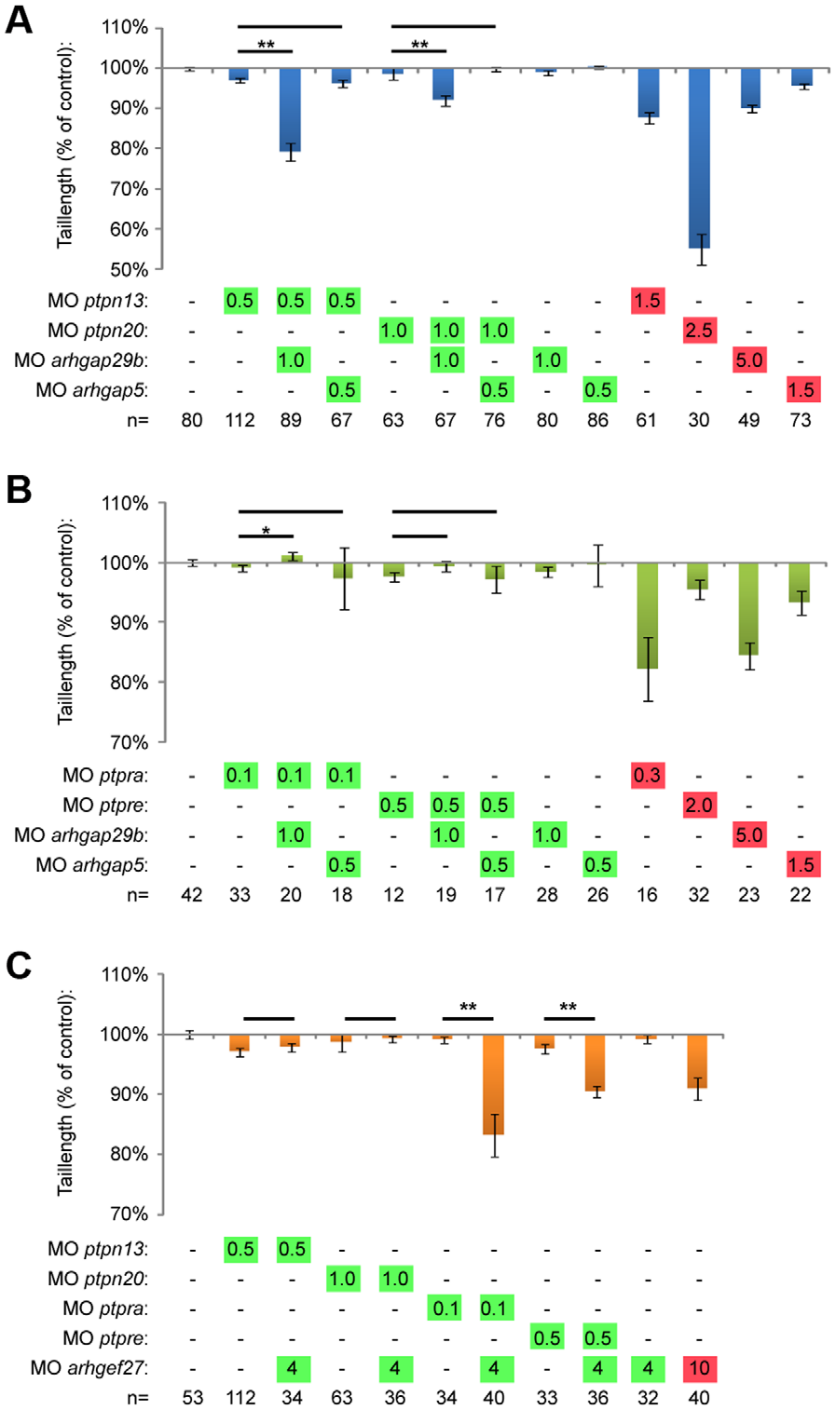
Arhgap29 and NGEF act downstream of distinct PTPs. (a) Low dose combined knockdowns of *ptpn13* or *ptpn20* and *arhgap29b* were performed by injecting indicated amounts of morpholino at the one cell stage. Tail lengths were measured at 3dpf and plotted. Co-knockdowns with *arhgap5* were included as a control. (b) Similar co-knockdowns as in (a) but with *ptpra* and *ptpre* knockdown instead of *ptpn13* and *ptpn20* knockdown. (c) Zebrafish embryos were micro-injected with morpholinos targeting the different phosphatases in low concentrations together with low dose *arhgef27* (*ngef*) morpholino. Embryos were grown to 3 dpf and tail lengths were determined and plotted as a percentage of non-injected control. All error bars are standard error of the mean. Student t-test was performed where indicated; no asterisk indicates P>0.05, * indicates 0.05>P>0.001 and ** indicates P<0.001.

To investigate whether PTP-BL and Ptpn20 interacted genetically with Arhgap29, we used combined low-dose knockdown of either *ptpn13* or *ptpn20* and *arhgap29b*. When we co-injected morpholinos targeting these genes, we were able to demonstrate that only combined low-dose knockdown of *ptpn13* or *ptpn20* with *arhgap29b* decreased tail length (Fig. 6a, Fig. S4). Combined knockdown with *arhgap5* did not induce a phenotype, suggesting that the Arhgap29b – PTP phenotype was specific and not a mere generic effect of combined knockdown with any Rho-GAP. Interestingly, when we performed combined low dose knockdown of *ptpra* or *ptpre* with *arhgap29b*, we did not see reconstitution of the shorter phenotype (Fig. 6b, Fig. S4).

To investigate functional interactions of the four PTPs with NGEF, we performed partial knockdowns of *ngef* and the different phosphatases. Analysis of the tail length at 3 dfp revealed that RPTPα and PTPε, but not PTP-BL and Ptpn20, interacted with NGEF (Fig. 6c, Fig. S4). These results are consistent with our hypothesis that RPTPα and PTPε are upstream activators of RhoA while PTP-BL and Ptpn20 inactivate RhoA. Combining low dose knockdown of either two activators (*ptpra*/*ptpre* and *ngef*) or in-activators (*ptpn13*/*ptpn20* and *arhgap29b*) results in a phenotype, whereas combined co-knockdown of an activator with an inactivator does not affect development.

## Discussion

**Figure 7:**
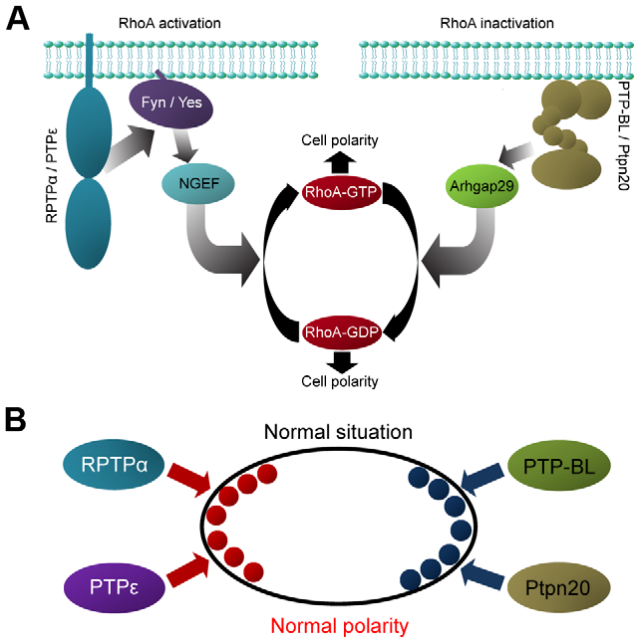
Model for PTP signaling in RhoA (in)activation and cell polarization. (a) RPTPα and PTPε are known activators of the SFKs, Fyn and Yes. Fyn and Yes either directly or indirectly activate NGEF by phosphorylation of Tyr-87 residue, increasing the specificity and activity of NGEF towards RhoA. PTP-BL and Ptpn20 likely indirectly activate Arhgap29 by either ensuring its recruitment or activation in order to inhibit RhoA activity. (b) Model for how enhanced and decreased RhoA activation may induce similar phenotypes. Assuming polarized distribution of RhoA-GTP (red dots) and RhoA-GDP (blue dots), either loss or increase of RhoA activation will result in loss of cell polarity (see text and Fig. S5 for further details).

Here, we describe four PTPs involved in regulating cell polarity in zebrafish C/E cell movements, RPTPα, PTPε, PTP-BL and Ptpn20 (Fig. 2 and 3). These phosphatases function in pairs, and have opposing effects on RhoA activation (Fig. 4, Fig. S3). Our data suggest a role for NGEF (ephexin1) and Arhgap29 (Parg1) as activators and inhibitors of RhoA activity in C/E movements downstream of PTP signaling. We propose a model (Fig. 7a), where RPTPα and PTPε dephosphorylate and activate the SFKs Fyn and Yes, which then leads to downstream activation of NGEF perhaps by direct phosphorylation of Tyr-87, resulting in RhoA activation. PTP-BL and Ptpn20 recruit and activate Arhgap29, leading to decreased RhoA activity downstream. Positive and negative effects of the PTPs on RhoA activation act in concert to mediate cell polarization which is at the basis of C/E cell movements.

Ptpn20 and Frmpd2 have been studied very little. In the Tiganis lab studies have been done on different isoforms of *PTPN20* using 5′ RACE [57]. They describe the identification of several isoforms, all consisting of the PTP domain only. Stenzel *et al.* report the basolateral targeting of Frmpd2 in epithelial cells and searched for different *FRMPD2* isoforms *in silico* [58]. No coding transcripts have been described so far spanning both the *FRMPD2* gene and the *PTPN20* gene. Our data clearly show that *frmpd2* and *ptpn20* sequences belong to the same gene (Fig. 1), but do not exclude the existence of the *ptpn20* isoforms described so far. We provide evidence that at least one additional *ptpn20* isoform exists, which is a paralogue of *ptpn13*. It would be interesting to investigate if indeed *frmpd2*, the PTP domain of *ptpn20* and the whole *ptpn20* as described here are separately expressed and have unique functions. Our results indicating that *ptpn13* and *ptpn20* are paralogues are not surprising, considering the high degree of conservation between their PTP domains. Their remarkable homology in sequence and structure clearly suggests a common ancestor. PTP-BL is a well-studied protein, and the identification of a paralogue brings a scala of interesting possibilities. Like PTP-BL, Ptpn20 is to be expected to act as an adaptor protein and participate in protein-protein interactions. *Ptpn13^(ΔP/ΔP)^* mice have a surprisingly mild phenotype [59], which could possibly be explained by partially redundant functions with *ptpn20*.

We demonstrate here the requirement of four phosphatases in normal C/E cell movements through their ability to regulate RhoA. Although RhoA is a well-known target of non-canonical Wnt signaling, an important signaling pathway in C/E cell movements, as well as other pathways controlling C/E, not much is known presently about the GEFs and GAPs controlling RhoA activity. Here we identified Arhgap29 (Parg1) and NGEF as Rho-GAP and Rho-GEF, respectively, for RhoA in C/E cell movements. Genetic interactions were established between *arhgap29* and *ptpn13*/*ptpn20*, two inhibitors of RhoA activity and between *ngef* and *ptpra*/*ptpre*, two activators of RhoA (Fig. 6). Knockdown of *ngef* or *arhgap29b* led to C/E cell movement defects mediated by impaired cell polarization (Fig. 5). Arhgap29 was originally identified as Parg1, PTP-BL interacting Rho-GAP [55] NGEF (ephexin1) is a well-established downstream component of Eph/ephrin signaling, and has been shown to play a role in axon pathfinding [60], [61], [62], [63].

Several lines of evidence presented here suggest a pathway controlled by tyrosine phosphatases regulating RhoA activity independently of the non-canonical Wnt signaling pathway. First we show that RPTPα, PTPε, PTP-BL and Ptpn20 have opposing effects on RhoA activation as RPTPα and PTPε knockdown induced defects can be rescued by co-injection of constitutively active RhoA, while PTP-BL and Ptpn20 knockdown induced defects can be rescued by co-injection of dominant negative RhoA in zebrafish embryos. Consistent with these data is the observation that RhoA is activated in HEK293T cells over-expressing RPTPα and inactivated in cells expressing PTP-BL (Fig. 4). Next we show genetic interactions of the Rho-GEF NGEF with RPTPα and PTPε, and the Rho-GAP Arhgap29b with PTP-BL and Ptpn20, resulting in a plausible explanation how RhoA activity can be altered downstream of these phosphatases (Fig. 6). Lastly we demonstrate by means of markers at the 1 somite and 7/8 somite stage as well as analysis of the cell shape and polarity during C/E cell movements that all genes involved indeed show defective C/E cell movements upon knockdown (Fig. 2, 5).

We present here the use of morpholino knockdown as a powerful tool for screening for components functioning within a single pathway, by co-injecting different morpholinos in suboptimal concentrations. The phenotypes demonstrated by knockdown of a single gene were reproduced by a second morpholino and/or rescued by co-injection of mRNA encoding the target gene, demonstrating specificity. Full knockdown of all the genes involved results in a phenotype. Full co-knockdowns of combinations of these genes often resulted in embryonic lethality, like co-knockdown of *ptpra* and *ptpre*, *ptpn13* and *ptpn20* or *ptpra* and *ptpn13* (data not shown). Suboptimal co-knockdowns allowed us to assess epigenetic interactions. Not all combinations of low dose morpholinos induced developmental defects, which enhanced confidence in our approach. Moreover, the inclusion of a control, *gap5*, that is not involved in C/E cell movements and does not result in an increased phenotype when co-injected at sub optimal doses, as opposed to *ngef* or *arhgap29b*, demonstrates the specificity of our approach. We use tail length at 3dpf as an easily quantifiable initial readout for the extent that the embryos are affected by combined low dose knockdowns. All genes described here were confirmed to be involved in C/E cell movements by analysis of their full knockdown phenotype using the appropriate markers at 1 somite and 7/8 somite stage, and analyzing cell polarity and cell shape in the presomitic mesoderm during C/E cell movements.

PTP-BL has previously been shown to be able to interact through its PDZ domains with PDZ binding proteins such as ephrin-B. Ephrin-B ligands have a PDZ binding motif at their C-terminus. Binding of PTP-BL has been suggested to regulate the dephosphorylation of the ephrin-B tyrosine 298 residue [50]. This residue was shown to be phosphorylated by SFKs, which has been verified *in vitro* by mass spectrometry analysis [64], and has been suggested to mediate the recruitment of Disheveled and RhoA. We have shown [45] that Fyn and Yes act downstream of RPTPα and PTPε, and mediate activation of RhoA, thus opposing the effect of PTP-BL and Ptpn20. It will be interesting to find out if Eph/ephrin-B signaling indeed link phosphatase signaling to downstream RhoA activation, which is also suggested by the involvement of NGEF, a downstream component of ephrin-B reverse signaling.

We propose a model for PTP regulated activation and inhibition of RhoA activity in C/E cell movements through NGEF and Arhgap29 (Fig. 7a). Activation of the SFKs Fyn and Yes by RPTPα and PTPε may directly lead to phosphorylation of NGEF on Tyr87 and conversion of RhoA-GDP to RhoA-GTP. PTP-BL and Ptpn20 recruit Arhgap29, leading to conversion of RhoA-GTP to RhoA-GDP. The two pairs of PTPs mediate activation and inactivation of RhoA, respectively. How both RhoA activation and inactivation contributes to C/E cell movements remains to be determined definitively. We hypothesize that asymmetric distribution of RhoA-GTP and RhoA-GDP over the leading and trailing edge of the cell is required for proper polarity and migration. Over-activation of RhoA or over-inhibition of RhoA will both result in loss of polarity, explaining why both knockdown of upstream activators like RPTPα and PTPε and inhibitors like PTP-BL and Ptpn20 lead to similar phenotypes (Fig. 7b). That inhibition and activation of a signaling pathway leads to similar phenotypes is not unprecedented. Noonan and LEOPARD mutations in Shp2 result in activation and inactivation of phosphatase activity, respectively, but both result in remarkably similar phenotypes in humans and zebrafish [44], [65]. Similarly, both inhibition and overexpression of Rok2 has been shown to induce similar phenotypes in zebrafish embryos [24]. Our data shows that RPTPα, PTPε, PTP-BL and Ptpn20 function in pairs, where low dose co-knockdown of *ptpra* and *ptpn13* resulted in a severe phenotype and co-knockdown of *ptpra* and *ptpn20* did not. Similarly, *ptpre* and *ptpn20*, but not *ptpn13* cooperated. To explain these results we suggest a model as depicted in Fig. 7b. We propose that RPTPα and PTPε act on one side of the cell as activators of RhoA, while PTP-BL and Ptpn20 act on the opposite side as inhibitors. Full knockdown of either one of the components, activating or inhibiting, will reduce RhoA-GTP or RhoA-GDP levels to such an extent that cell polarity will be lost (cf. Fig. S5). Low dose knockdown of a single component mildly reduces RhoA-GTP or RhoA-GDP levels, but does not result in loss of polarity and defective C/E cell movements, because of normal signaling on the opposing side. Combined low dose knockdown however, will lead to reduced activation of RhoA on one side and reduced inhibition of RhoA on the other side, resulting in loss of polarity and hence C/E cell movement defects (Fig. S5). We speculate that upstream activation of the different PTPs, subcellular localization of the PTPs and their target proteins, substrate specificity and cell type specific expression may play a role in fine-tuning of the regulation of RhoA activity, possibly explaining the specificity of the combined knockdowns, i.e. why combined knockdown of *ptpra* and *ptpn13*, but not *ptpra* and *ptpn20*, induced C/E cell movement defects.

## Methods

### Ethics statement

Only wild type embryos up to 3 dpf were used for these experiments, which do not require approval of the animal experiments committee according to national and European law.

### Zebrafish maintenance and *in situ* hybridization and microinjection

Zebrafish were kept and the embryos were staged as described before [66]. *In situ* hybridizations were done essentially as described [67] using probes specific for *dlx3* (currently known as *dlx3b*), *hgg1* (currently known as *ctsl1b*), *krox20* and *myod* as described earlier [44], [54]. Zebrafish were injected at the one cell stage in the cell with. Needles were calibrated to dispense 1 nanoliter volumes. Embryos were kept in E3 medium at 28.5°C. A considerable part of our results are based on measuring tail length as readout for the severity of observed phenotypes. To ensure correct interpretation of the results, we injected all morpholino and RNA constructs into the cell at the 1 cell stage, as opposed to the yolk, to ensure equal delivery of injected cargo. Since variation in volumes injected may affect the observed phenotype, special attention was paid to calibrating every needle used, and verifying amounts injected after each injection series. To minimize variation, injection conditions that were compared were injected in a single batch of embryos.

### Morpholinos and RNA

Morpholinos for *ptpra*, *ptprea* and *ptpreb* have been described before [46]. Morpholinos targeting *ptpn13*, *ptpn20*, *arhgap29b* and *arhgap5* were designed as splice donor targeting, using the following sequences: MO *ptpn13*: 5′CTCTCTCTCTCACCTGGACGTCTTT′3; MO *ptpn20*: 5′AGAATAAGCTTACACAGAGGTGGGG′3; MO *arhgap29b*: 5′GTGCTATTGTACCTGTGCAGATGTG′3 and MO *arhgap5*: 5′GACGGGTCTCCTTATTCTTGGCCAT′3. *Ptpn13* RNA was transcribed from full length mouse cDNA kindly provided by Wiljan Hendriks (Department of Cell Biology, Radboud University Nijmegen Medical Centre, Nijmegen, The Netherlands).

### Westernblot and IP

Zebrafish embryos were microinjected and raised in standard conditions. At 28hpf: whole embryos were lysed inside the chorion in buffer containing 50 mM Tris, pH 7.5, 150 mM NaCl, 1 mM EDTA, 1 mM sodium orthovanadate, 1% Nonidet P-40, 0.1% sodium deoxycholate, protease inhibitor mixture (Complete Mini, Roche Diagnostics) and vanadate, using a bioruptor and 30 µl lysisbuffer for each embryo. Lysates were spun down and 4× sample buffer was added to supernatant; Samples were run on SDS-PAGE gel (15%) and transferred to PVDF membrane. After transfer the membrane was stained with Coomassie Blue stain to verify equal loading of the lysates. Subsequently the PVDF membrane was blocked with 5% BSA and then incubated with the corresponding antibodies targeting pY-87-ephexin1 (EP2841 rabbit polyclonal; ECM Biosciences - Versailles, KY, USA) or Actin (A5060 Anti-Actin 20–33 rabbit polyclonal; Sigma Aldrich – St. Louis, MO, USA) followed by the horseradish peroxidase conjugated secondary antibody. The membranes were subjected to detection by enhanced chemiluminescence.

### Confocal microscopy

To achieve ubiquitous fluorescent membrane labeling, the embryos were injected at 1 cell stage with 20 pg of mRNA encoding membrane-citrine (an YFP variant with a C-terminal fusion of the Ras membrane-localization sequence [CAAX]). To visualize the cell shape in the presomitic mesoderm, membrane-citrine expressing live embryos were mounted in 0.75% soft agarose at the dorsal side in glass bottomed Petri dishes. Using a SP2 Leica confocal microscope the presomitic mesoderm was imaged using a 40× oil objective. Images were processed in ImageJ and, analysis of cell length-to-width ratio and angular deviation was performed by the Shape_Descriptor1u plugin [68].

### Tail length assay and statistics

Embryos were microinjected at the one cell stage and grown under standard conditions to 3dpf. Pictures were taken at identical magnification and tail lengths were measured using ImageJ software. All tail lengths were calculated as percentage of non-injected control embryos from the same clutch. Comparisons were done between different injection conditions within the same clutch. Results of at least three individual experiments were pooled and tail lengths were plotted as percentages deviating from 100%. We compared tail lengths of 2 and 3 dpf embryos, measured from the border of the yolk-yolk extension to the tip of the tail and found that tail length increases about 6% from day 2 to day 3. In our experience, injection of morpholino or mRNA inducing phenotypes generally induces a delay of approximately 1 hour maximum at the 1 somite stage. Error bars represent S.E.M. in al graphs. Two tailed student t-tests assuming unequal variance were performed to compare individual injection conditions. Total number of samples are indicated in figures, throughout figures, P-values are represented by no asterix (P>0.05), * (0.05>P>0.001) or ** (P<0.001), individual P-values are indicated in the figure legends.

## Supporting Information

Figure S1 **Protein tyrosine phosphatase genes in five fish species identified by blasting.** Fish orthologs of all PTP encoding genes were identified by BLASTing the PTP domains of every single human PTP gene against the 5 respective zebrafish genomes. Indicated are genes already annotated in Ensembl (green), or 2 genes already annotated (light blue), 1 gene annotated, 1 additional one found by blasting (dark blue), none annotated, 1 identified by blasting (orange), none annotated and 2 identified by blasting (purple) or none annotated and none identified (red).
(TIF)

Figure S2 ***Ptpn13*** **knockdown phenotype can be rescued by co-injection of** ***ptpn13*** **mRNA.** Zebrafish embryos were microinjected at the one cell stage with MO *ptpn13* alone or in combination with mouse *ptpn13* mRNA. Fish were grown to 3dpf and tail lengths were measured. Average tail length relative to non-injected control is plotted. All error bars are standard error of the mean. Student t-test was performed where indicated; ** indicates P<0.001.
(TIF)

Figure S3 **PTP knockdowns are rescued by active or dominant negative RhoA.** Embryos were micro-injected at the one cell stage using morpholinos (high concentration) targeting the indicated genes together with no RNA, RNA encoding constitutively active RhoA (3 pg/embryo) or RNA encoding dominant negative RhoA (20 pg/embryo). Fish were grown to 3dpf and pictures were taken; representative fish for each condition are shown.
(TIF)

Figure S4 **Arhgap29 and NGEF act downstream of distinct PTPs.** Low dose combined knockdowns of *ptpn13*, *ptpn20*, *ptpra*, or *ptpre* and *arhgap29b* or *arhgef27* (*ngef*) were performed by injecting indicated amounts of morpholino at the one cell stage. Fish were grown to 3dpf and pictures were taken; representative embryos for each condition are shown. Co-knockdowns with *arhgap5* were included as a control.
(TIF)

Figure S5 **Model for low dose PTP co-knockdown-induced defects.** In the normal situation RPTPα and PTPε activate RhoA one side of the cell and RhoA activity is inhibited on the opposing side by PTP-BL and Ptpn20. Normal RhoA activation and inhibition of RhoA is indicated by thick red and blue arrows, respectively. RhoA-GTP is schematically indicated by red dot, Rho-GDP by blue dot. Deletion of an inactivator (PTP-BL) or activator (RPTPα) – indicated by strike-through - results in reduced RhoA-GDP or RhoA-GTP on one side of the cell, respectively, and hence loss of polarity. Low dose knockdown of one of the PTPs (thin arrows) results in small differences in RhoA-GTP/RhoA-GDP distribution that do not affect cell polarization. Partial knockdown of both activators (or both inactivators, not shown) will result in severe changes in RhoA activation on one side of the cell and hence disturb cell polarization. Partial activation and partial inhibition of RhoA may lead to reduction of RhoA-GTP on one side of the cell and reduction of RhoA-GDP on the other side of the cell, hence disturbing cell polarization. Together, this model explains how two pairs of PTPs with opposing effects on RhoA activation act in concert to maintain cell polarization that is at the basis of convergence and extension cell movements during zebrafish gastrulation.
(TIF)

Table S1 Non-annotated PTP genes in four fish species identified by blasting. The PTP domains of human phosphatases were blasted against the genomes of fugu, medaka, tetraodon and stickleback. All PTP encoding genes identified not previously annotated as being a PTP encoding gene are listed here with corresponding gene name appended with a or b in case of gene duplication.
(DOC)

## References

[1] Keller R (2002). Shaping the vertebrate body plan by polarized embryonic cell movements.. Science.

[2] Warga RM, Kimmel CB (1990). Cell movements during epiboly and gastrulation in zebrafish.. Development.

[3] Heisenberg CP, Tada M, Rauch GJ, Saude L, Concha ML (2000). Silberblick/Wnt11 mediates convergent extension movements during zebrafish gastrulation.. Nature.

[4] Keys DN, Levine M, Harland RM, Wallingford JB (2002). Control of intercalation is cell-autonomous in the notochord of Ciona intestinalis.. Dev Biol.

[5] Wallingford JB, Fraser SE, Harland RM (2002). Convergent extension: the molecular control of polarized cell movement during embryonic development.. DevCell.

[6] Wallingford JB, Rowning BA, Vogeli KM, Rothbacher U, Fraser SE (2000). Dishevelled controls cell polarity during Xenopus gastrulation.. Nature.

[7] Ybot-Gonzalez P, Gaston-Massuet C, Girdler G, Klingensmith J, Arkell R (2007). Neural plate morphogenesis during mouse neurulation is regulated by antagonism of Bmp signalling.. Development.

[8] Wang Y, Nathans J (2007). Tissue/planar cell polarity in vertebrates: new insights and new questions.. Development.

[9] Murdoch JN, Henderson DJ, Doudney K, Gaston-Massuet C, Phillips HM (2003). Disruption of scribble (Scrb1) causes severe neural tube defects in the circletail mouse.. Hum Mol Genet.

[10] Myers DC, Sepich DS, Solnica-Krezel L (2002). Bmp activity gradient regulates convergent extension during zebrafish gastrulation.. Dev Biol.

[11] von der Hardt S, Bakkers J, Inbal A, Carvalho L, Solnica-Krezel L (2007). The Bmp gradient of the zebrafish gastrula guides migrating lateral cells by regulating cell-cell adhesion.. Curr Biol.

[12] Ataliotis P, Symes K, Chou MM, Ho L, Mercola M (1995). PDGF signalling is required for gastrulation of Xenopus laevis.. Development.

[13] Ghil JS, Chung HM (1999). Evidence that platelet derived growth factor (PDGF) action is required for mesoderm patterning in early amphibian (Xenopus laevis) embryogenesis.. Int J Dev Biol.

[14] Symes K, Mercola M (1996). Embryonic mesoderm cells spread in response to platelet-derived growth factor and signaling by phosphatidylinositol 3-kinase.. Proc Natl Acad Sci U S A.

[15] Conway G, Margoliath A, Wong-Madden S, Roberts RJ, Gilbert W (1997). Jak1 kinase is required for cell migrations and anterior specification in zebrafish embryos.. Proc Natl Acad Sci U S A.

[16] Miyagi C, Yamashita S, Ohba Y, Yoshizaki H, Matsuda M (2004). STAT3 noncell-autonomously controls planar cell polarity during zebrafish convergence and extension.. J Cell Biol.

[17] Chan J, Mably JD, Serluca FC, Chen JN, Goldstein NB (2001). Morphogenesis of prechordal plate and notochord requires intact Eph/ephrin B signaling.. Dev Biol.

[18] Jones TL, Chong LD, Kim J, Xu RH, Kung HF (1998). Loss of cell adhesion in Xenopus laevis embryos mediated by the cytoplasmic domain of XLerk, an erythropoietin-producing hepatocellular ligand.. Proc Natl Acad Sci U S A.

[19] Oates AC, Lackmann M, Power MA, Brennan C, Down LM (1999). An early developmental role for eph-ephrin interaction during vertebrate gastrulation.. Mech Dev.

[20] Hammerschmidt M, Pelegri F, Mullins MC, Kane DA, Brand M (1996). Mutations affecting morphogenesis during gastrulation and tail formation in the zebrafish, Danio rerio.. Development.

[21] Heisenberg CP, Brand M, Jiang YJ, Warga RM, Beuchle D (1996). Genes involved in forebrain development in the zebrafish, Danio rerio.. Development.

[22] Jessen JR, Topczewski J, Bingham S, Sepich DS, Marlow F (2002). Zebrafish trilobite identifies new roles for Strabismus in gastrulation and neuronal movements.. Nat Cell Biol.

[23] Kilian B, Mansukoski H, Barbosa FC, Ulrich F, Tada M (2003). The role of Ppt/Wnt5 in regulating cell shape and movement during zebrafish gastrulation.. Mech Dev.

[24] Marlow F, Topczewski J, Sepich D, Solnica-Krezel L (2002). Zebrafish Rho kinase 2 acts downstream of Wnt11 to mediate cell polarity and effective convergence and extension movements.. CurrBiol.

[25] Sepich DS, Myers DC, Short R, Topczewski J, Marlow F (2000). Role of the zebrafish trilobite locus in gastrulation movements of convergence and extension.. Genesis.

[26] Solnica-Krezel L, Stemple DL, Mountcastle-Shah E, Rangini Z, Neuhauss SC (1996). Mutations affecting cell fates and cellular rearrangements during gastrulation in zebrafish.. Development.

[27] Topczewski J, Sepich DS, Myers DC, Walker C, Amores A (2001). The zebrafish glypican knypek controls cell polarity during gastrulation movements of convergent extension.. Dev Cell.

[28] Chae J, Kim MJ, Goo JH, Collier S, Gubb D (1999). The Drosophila tissue polarity gene starry night encodes a member of the protocadherin family.. Development.

[29] Feiguin F, Hannus M, Mlodzik M, Eaton S (2001). The ankyrin repeat protein Diego mediates Frizzled-dependent planar polarization.. Dev Cell.

[30] Gubb D, Green C, Huen D, Coulson D, Johnson G (1999). The balance between isoforms of the prickle LIM domain protein is critical for planar polarity in Drosophila imaginal discs.. Genes Dev.

[31] Klingensmith J, Noll E, Perrimon N (1989). The segment polarity phenotype of Drosophila involves differential tendencies toward transformation and cell death.. Dev Biol.

[32] Taylor J, Abramova N, Charlton J, Adler PN (1998). Van Gogh: a new Drosophila tissue polarity gene.. Genetics.

[33] Vinson CR, Conover S, Adler PN (1989). A Drosophila tissue polarity locus encodes a protein containing seven potential transmembrane domains.. Nature.

[34] Jenny A, Darken RS, Wilson PA, Mlodzik M (2003). Prickle and Strabismus form a functional complex to generate a correct axis during planar cell polarity signaling.. EMBO J.

[35] Montcouquiol M, Kelley MW (2003). Planar and vertical signals control cellular differentiation and patterning in the mammalian cochlea.. J Neurosci.

[36] Boutros M, Mlodzik M (1999). Dishevelled: at the crossroads of divergent intracellular signaling pathways.. Mech Dev.

[37] Boutros M, Paricio N, Strutt DI, Mlodzik M (1998). Dishevelled activates JNK and discriminates between JNK pathways in planar polarity and wingless signaling.. Cell.

[38] Strutt DI, Weber U, Mlodzik M (1997). The role of RhoA in tissue polarity and Frizzled signalling.. Nature.

[39] Dunah AW, Hueske E, Wyszynski M, Hoogenraad CC, Jaworski J (2005). LAR receptor protein tyrosine phosphatases in the development and maintenance of excitatory synapses.. Nat Neurosci.

[40] Elchebly M, Wagner J, Kennedy TE, Lanctot C, Michaliszyn E (1999). Neuroendocrine dysplasia in mice lacking protein tyrosine phosphatase sigma.. Nat Genet.

[41] Wallace MJ, Batt J, Fladd CA, Henderson JT, Skarnes W (1999). Neuronal defects and posterior pituitary hypoplasia in mice lacking the receptor tyrosine phosphatase PTPsigma.. Nat Genet.

[42] Saunders AE, Johnson P (2010). Modulation of immune cell signalling by the leukocyte common tyrosine phosphatase, CD45.. Cell Signal.

[43] Tartaglia M, Mehler EL, Goldberg R, Zampino G, Brunner HG (2001). Mutations in PTPN11, encoding the protein tyrosine phosphatase SHP-2, cause Noonan syndrome.. NatGenet.

[44] Jopling C, van Geemen D, den Hertog J (2007). Shp2 knockdown and Noonan/LEOPARD mutant Shp2-induced gastrulation defects.. PLoS Genet.

[45] van Eekelen M, Runtuwene V, Overvoorde J, den Hertog J (2010). RPTPalpha and PTPepsilon signaling via Fyn/Yes and RhoA is essential for zebrafish convergence and extension cell movements during gastrulation.. Dev Biol.

[46] van Eekelen M, Overvoorde J, van Rooijen C, den Hertog J (2010). Identification and expression of the family of classical protein-tyrosine phosphatases in zebrafish.. PLoS One.

[47] Li Y, Kanki H, Hachiya T, Ohyama T, Irie S (2000). Negative regulation of Fas-mediated apoptosis by FAP-1 in human cancer cells.. Int J Cancer.

[48] Ungefroren H, Voss M, Jansen M, Roeder C, Henne-Bruns D (1998). Human pancreatic adenocarcinomas express Fas and Fas ligand yet are resistant to Fas-mediated apoptosis.. Cancer Res.

[49] Lin D, Gish GD, Songyang Z, Pawson T (1999). The carboxyl terminus of B class ephrins constitutes a PDZ domain binding motif.. J Biol Chem.

[50] Palmer A, Zimmer M, Erdmann KS, Eulenburg V, Porthin A (2002). EphrinB phosphorylation and reverse signaling: regulation by Src kinases and PTP-BL phosphatase.. Mol Cell.

[51] Hatta K, Takahashi Y (1996). Secondary axis induction by heterospecific organizers in zebrafish.. Developmental dynamics : an official publication of the American Association of Anatomists.

[52] Li H, Marijanovic I, Kronenberg MS, Erceg I, Stover ML (2008). Expression and function of Dlx genes in the osteoblast lineage.. Developmental biology.

[53] Jopling C, den Hertog J (2005). Fyn/Yes and non-canonical Wnt signalling converge on RhoA in vertebrate gastrulation cell movements.. EMBO Rep.

[54] Jopling C, Hertog J (2007). Essential role for Csk upstream of Fyn and Yes in zebrafish gastrulation.. Mech Dev.

[55] Saras J, Franzen P, Aspenstrom P, Hellman U, Gonez LJ (1997). A novel GTPase-activating protein for Rho interacts with a PDZ domain of the protein-tyrosine phosphatase PTPL1.. J Biol Chem.

[56] Zhang Y, Sawada T, Jing X, Yokote H, Yan X (2007). Regulation of ephexin1, a guanine nucleotide exchange factor of Rho family GTPases, by fibroblast growth factor receptor-mediated tyrosine phosphorylation.. J Biol Chem.

[57] Fodero-Tavoletti MT, Hardy MP, Cornell B, Katsis F, Sadek CM (2005). Protein tyrosine phosphatase hPTPN20a is targeted to sites of actin polymerization.. Biochem J.

[58] Stenzel N, Fetzer CP, Heumann R, Erdmann KS (2009). PDZ-domain-directed basolateral targeting of the peripheral membrane protein FRMPD2 in epithelial cells.. J Cell Sci.

[59] Wansink DG, Peters W, Schaafsma I, Sutmuller RP, Oerlemans F (2004). Mild impairment of motor nerve repair in mice lacking PTP-BL tyrosine phosphatase activity.. Physiol Genomics.

[60] Egea J, Nissen UV, Dufour A, Sahin M, Greer P (2005). Regulation of EphA 4 kinase activity is required for a subset of axon guidance decisions suggesting a key role for receptor clustering in Eph function.. Neuron.

[61] Knoll B, Drescher U (2004). Src family kinases are involved in EphA receptor-mediated retinal axon guidance.. J Neurosci.

[62] Sahin M, Greer PL, Lin MZ, Poucher H, Eberhart J (2005). Eph-dependent tyrosine phosphorylation of ephexin1 modulates growth cone collapse.. Neuron.

[63] Shamah SM, Lin MZ, Goldberg JL, Estrach S, Sahin M (2001). EphA receptors regulate growth cone dynamics through the novel guanine nucleotide exchange factor ephexin.. Cell.

[64] Jorgensen C, Sherman A, Chen GI, Pasculescu A, Poliakov A (2009). Cell-specific information processing in segregating populations of Eph receptor ephrin-expressing cells.. Science.

[65] Edouard T, Montagner A, Dance M, Conte F, Yart A (2007). How do Shp2 mutations that oppositely influence its biochemical activity result in syndromes with overlapping symptoms?. Cell Mol Life Sci.

[66] Westerfield M (1995). The Zebrafish Book.

[67] Thisse C, Thisse B, Schilling TF, Postlethwait JH (1993). Structure of the zebrafish snail1 gene and its expression in wild-type, spadetail and no tail mutant embryos.. Development.

[68] Syverud K, Chinga G, Johnsen PO, Leirset I, Wiik K (2007). Analysis of lint particles from full-scale printing trials.. APPITA.

