# Supplementary figures and images for: Pair-Wise Regulation of Convergence and Extension Cell Movements by Four Phosphatases via RhoA
Source: PLoS One. 2012 Apr 24;7(4):e35913. doi: 10.1371/journal.pone.0035913 (PMC3335823; doi:10.1371/journal.pone.0035913)

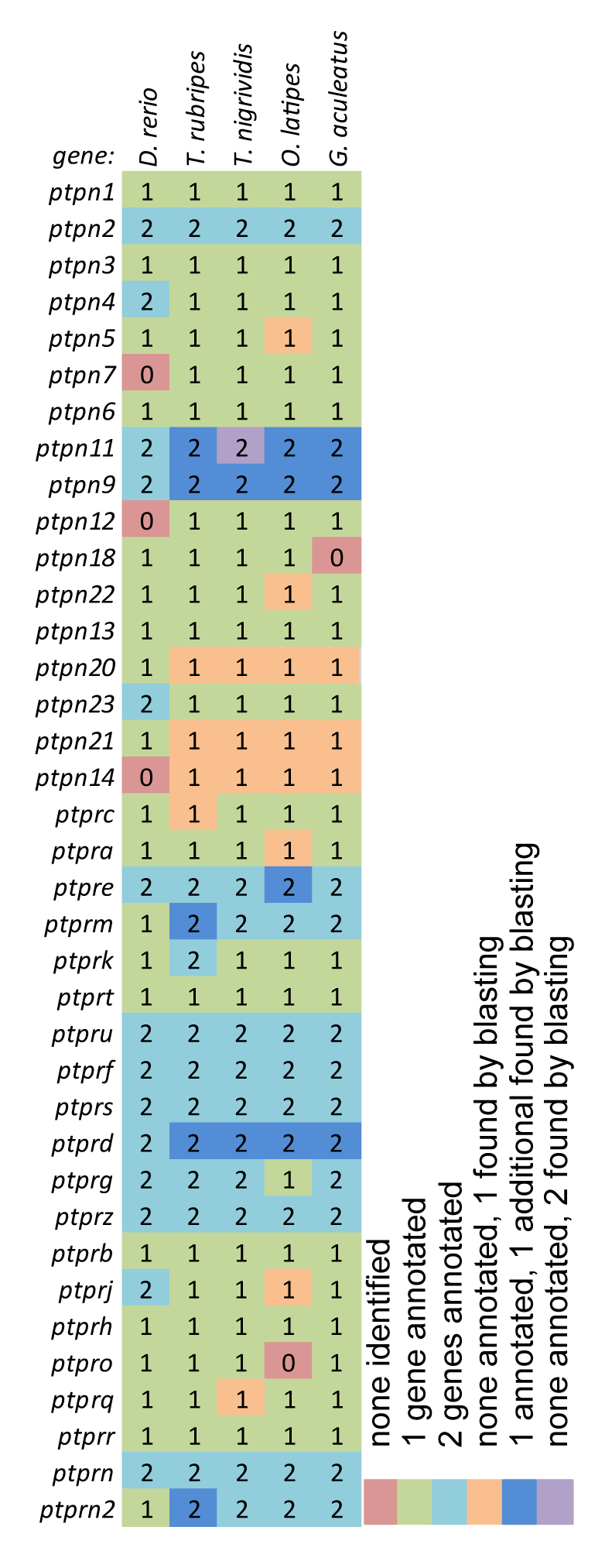

Supplement: Figure S1 — Protein tyrosine phosphatase genes in five fish species identified by blasting. Fish orthologs of all PTP encoding genes were identified by BLASTing the PTP domains of every single human PTP gene against the 5 respective zebrafish genomes. Indicated are genes already annotated in Ensembl (green), or 2 genes already annotated (light blue), 1 gene annotated, 1 additional one found by blasting (dark blue), none annotated, 1 identified by blasting (orange), none annotated and 2 identified by blasting (purple) or none annotated and none identified (red). (TIF) [file pone.0035913.s001.tif]

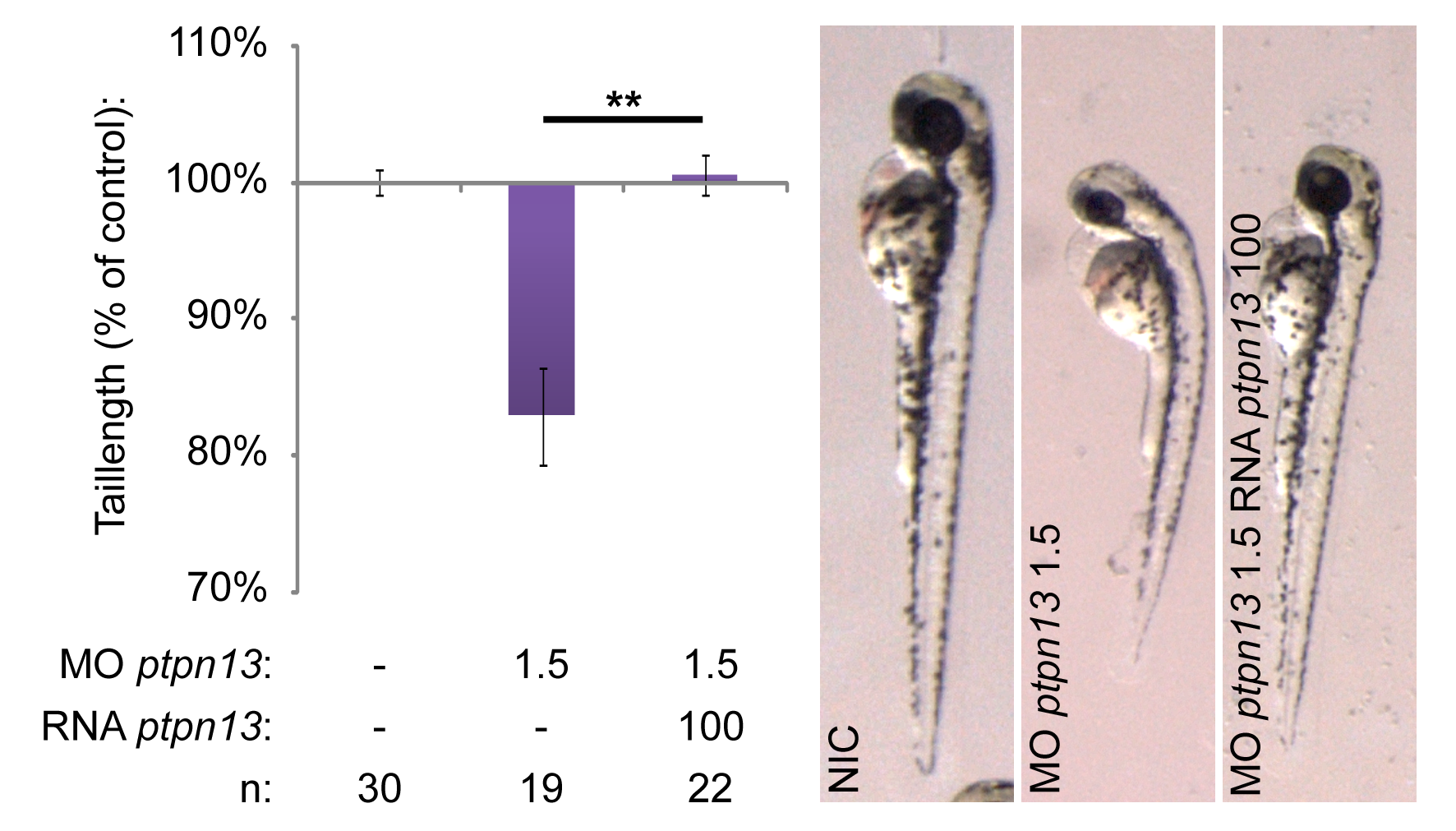

Supplement: Figure S2 — Ptpn13 knockdown phenotype can be rescued by co-injection of ptpn13 mRNA. Zebrafish embryos were microinjected at the one cell stage with MO ptpn13 alone or in combination with mouse ptpn13 mRNA. Fish were grown to 3dpf and tail lengths were measured. Average tail length relative to non-injected control is plotted. All error bars are standard error of the mean. Student t-test was performed where indicated; ** indicates P<0.001. (TIF) [file pone.0035913.s002.tif]

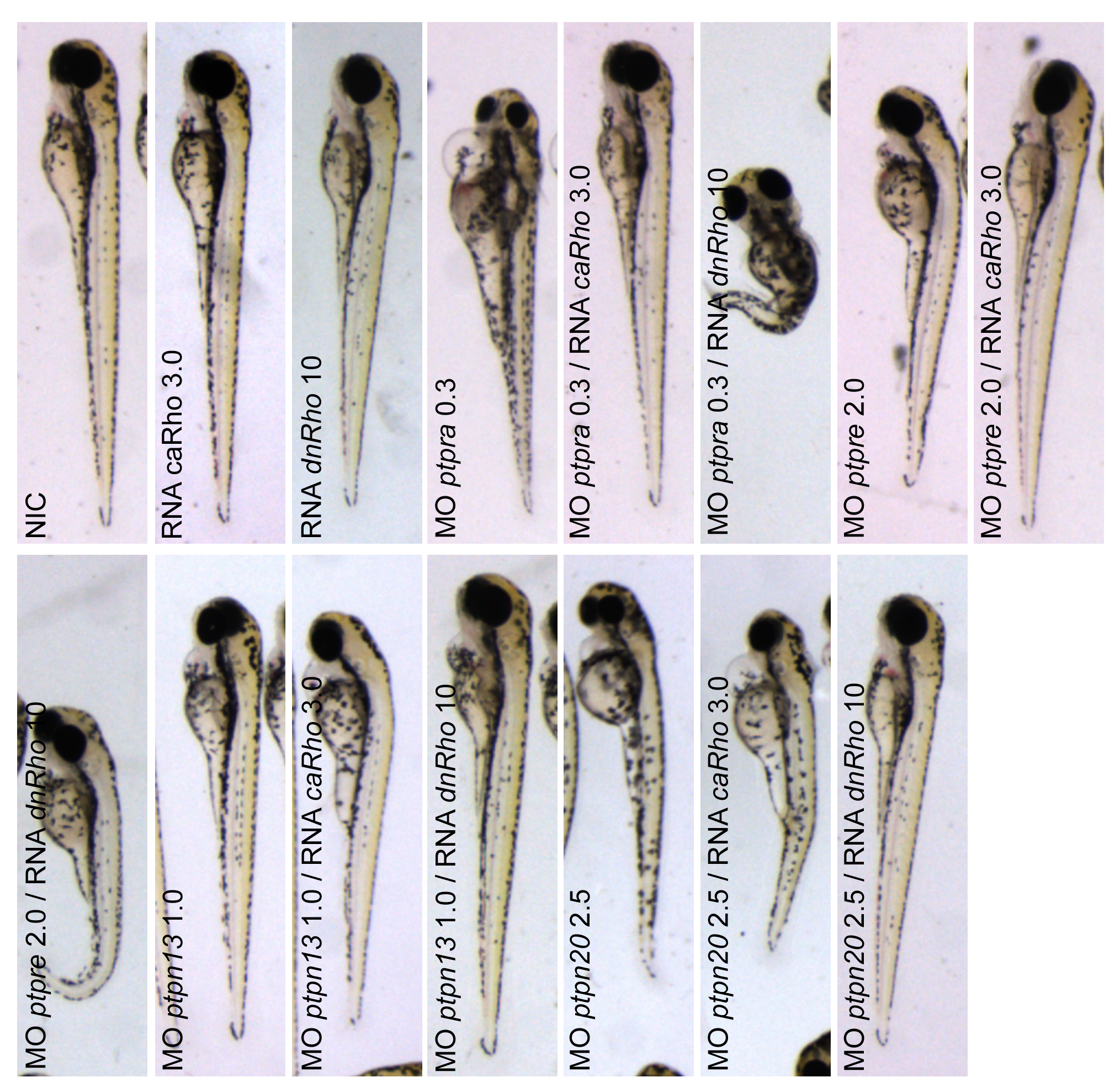

Supplement: Figure S3 — PTP knockdowns are rescued by active or dominant negative RhoA. Embryos were micro-injected at the one cell stage using morpholinos (high concentration) targeting the indicated genes together with no RNA, RNA encoding constitutively active RhoA (3 pg/embryo) or RNA encoding dominant negative RhoA (20 pg/embryo). Fish were grown to 3dpf and pictures were taken; representative fish for each condition are shown. (TIF) [file pone.0035913.s003.tif]

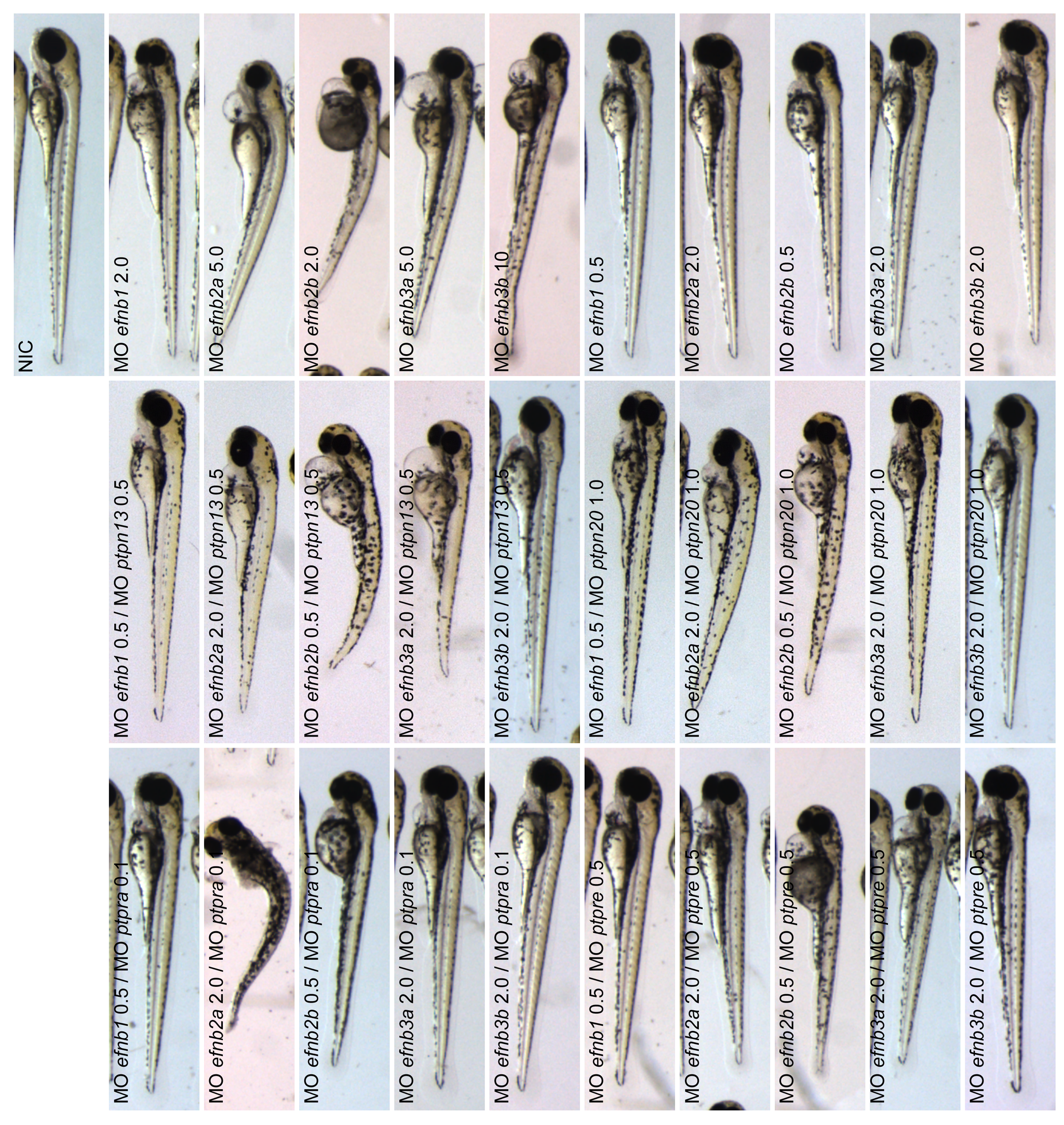

Supplement: Figure S4 — Arhgap29 and NGEF act downstream of distinct PTPs. Low dose combined knockdowns of ptpn13, ptpn20, ptpra, or ptpre and arhgap29b or arhgef27 (ngef) were performed by injecting indicated amounts of morpholino at the one cell stage. Fish were grown to 3dpf and pictures were taken; representative embryos for each condition are shown. Co-knockdowns with arhgap5 were included as a control. (TIF) [file pone.0035913.s004.tif]

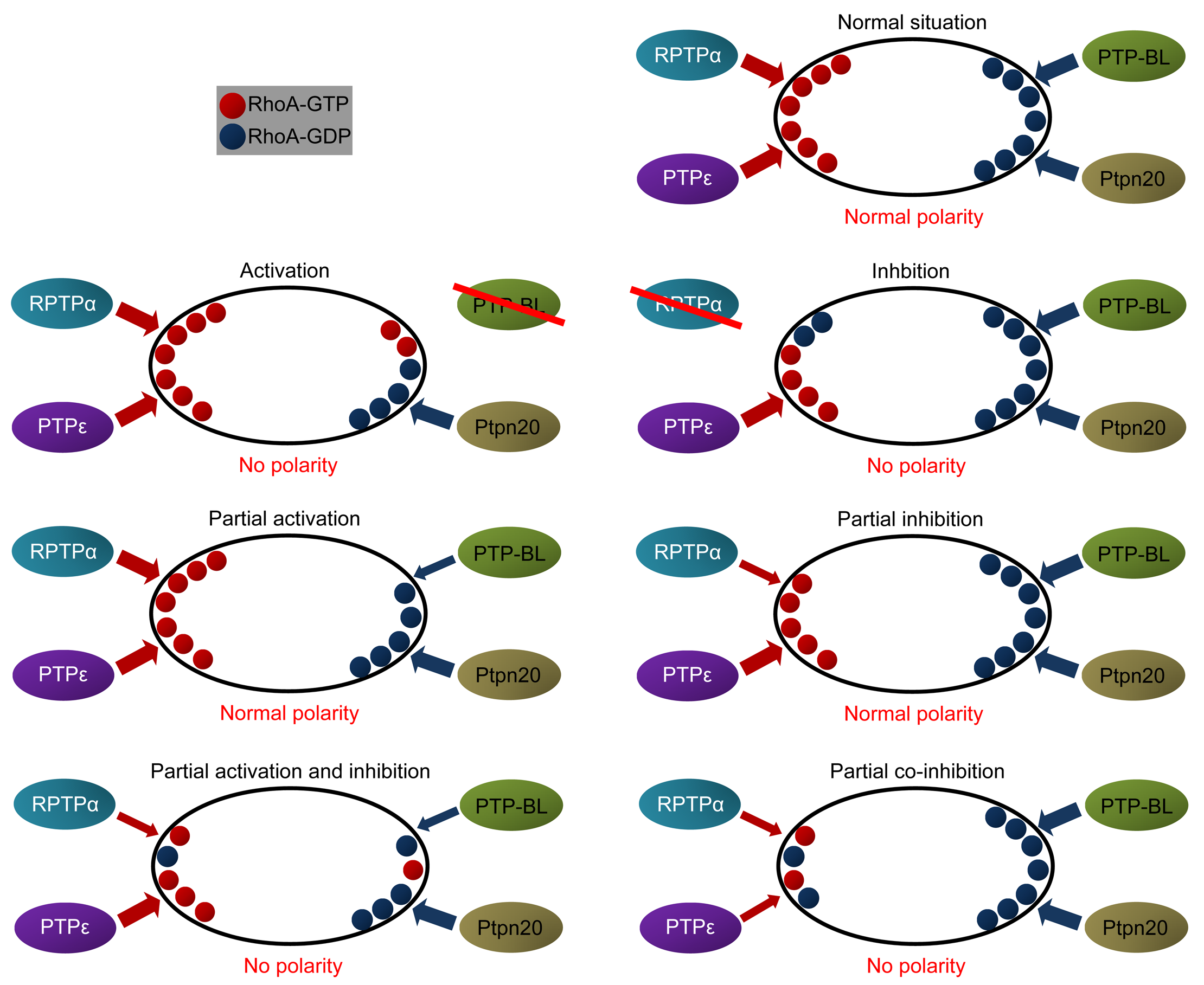

Supplement: Figure S5 — Model for low dose PTP co-knockdown-induced defects. In the normal situation RPTPα and PTPε activate RhoA one side of the cell and RhoA activity is inhibited on the opposing side by PTP-BL and Ptpn20. Normal RhoA activation and inhibition of RhoA is indicated by thick red and blue arrows, respectively. RhoA-GTP is schematically indicated by red dot, Rho-GDP by blue dot. Deletion of an inactivator (PTP-BL) or activator (RPTPα) – indicated by strike-through - results in reduced RhoA-GDP or RhoA-GTP on one side of the cell, respectively, and hence loss of polarity. Low dose knockdown of one of the PTPs (thin arrows) results in small differences in RhoA-GTP/RhoA-GDP distribution that do not affect cell polarization. Partial knockdown of both activators (or both inactivators, not shown) will result in severe changes in RhoA activation on one side of the cell and hence disturb cell polarization. Partial activation and partial inhibition of RhoA may lead to reduction of RhoA-GTP on one side of the cell and reduction of RhoA-GDP on the other side of the cell, hence disturbing cell polarization. Together, this model explains how two pairs of PTPs with opposing effects on RhoA activation act in concert to maintain cell polarization that is at the basis of convergence and extension cell movements during zebrafish gastrulation. (TIF) [file pone.0035913.s005.tif]
