# Supplementary material for: Pair-Wise Regulation of Convergence and Extension Cell Movements by Four Phosphatases via RhoA
Source: PLoS One. 2012 Apr 24;7(4):e35913. doi: 10.1371/journal.pone.0035913 (PMC3335823; doi:10.1371/journal.pone.0035913)
Supplement: Table S1 — Non-annotated PTP genes in four fish species identified by blasting. The PTP domains of human phosphatases were blasted against the genomes of fugu, medaka, tetraodon and stickleback. All PTP encoding genes identified not previously annotated as being a PTP encoding gene are listed here with corresponding gene name appended with a or b in case of gene duplication. (DOC) [file pone.0035913.s006.doc]

**Table S1. Non-annotated PTP genes in four fish species identified by blasting.**

| Species: | Gene: | Location: |
| --- | --- | --- |
| *T. Rubripes* | ptpn11b | ENSTRUG00000006764 |
|  | ptpn9b | ENSTRUG00000004960 |
|  | ptpn20 | ENSTRUG00000012682 |
|  | ptpn21 | ENSTRUG00000013692 |
|  | ptpn14 | ENSTRUG00000014100 |
|  | ptprc | NP_001027788.1 |
|  | ptprmb | scaffold_297: 283,607-287,768 |
|  | ptprdb | scaffold_11124 |
|  | ptprn2b | scaffold_9445 |
| *T. Nigrividis* | ptpn11a | GSTENT10016339001 |
|  | ptpn11b | ENSTNIG00000010883 |
|  | ptpn9b | ENSTNIG00000017817 |
|  | ptpn20 | ENSTNIG00000003885 |
|  | ptpn21 | ENSTNIG00000017244 |
|  | ptpn14 | ENSTNIG00000016380 |
|  | ptprdb | ENSTNIG00000004098 |
|  | ptprq | GSTENG10005433001 |
| *O. Latipes* | ptpn11b | ENSORLG00000000613 |
|  | ptpn9b | ENSORLG00000005257 |
|  | ptpn22 | scaffold12_contig11260 |
|  | ptpn20 | FRMPD2 |
|  | ptpn21 | ENSORLG00000012974 |
|  | ptpn14 | ENSORLG00000014908 |
|  | ptpra | Q76K58_ORYLA |
|  | ptpreb | Q76K57_ORYLA |
|  | ptprdb | ENSORLG00000004685 |
|  | ptprj | ENSORLG00000017385 |
| *G. Aculeatus* | ptpn11b | ENSGACG00000004807 |
|  | ptpn9b | ENSGACG00000016107 |
|  | ptpn20 | ENSGACG00000007126 |
|  | ptpn21 | ENSGACG00000010542 |
|  | ptpn14 | ENSGACG00000010112 |
|  | ptprdb | ENSGACG00000015921 |
